# Efficacy of Phytochemicals Derived from *Avicennia officinalis* for the Management of COVID-19: A Combined In Silico and Biochemical Study

**DOI:** 10.3390/molecules26082210

**Published:** 2021-04-12

**Authors:** Shafi Mahmud, Gobindo Kumar Paul, Mirola Afroze, Shirmin Islam, Swagota Briti Ray Gupt, Mamudul Hasan Razu, Suvro Biswas, Shahriar Zaman, Md. Salah Uddin, Mala Khan, Nunzio Antonio Cacciola, Talha Bin Emran, Md. Abu Saleh, Raffaele Capasso, Jesus Simal-Gandara

**Affiliations:** 1 Microbiology Laboratory, Department of Genetic Engineering and Biotechnology, University of Rajshahi, Rajshahi 6205, Bangladesh; shafimahmudfz@gmail.com (S.M.); gobindokumar38@gmail.com (G.K.P.); buligeb1127@gmail.com (S.I.); szaman@ru.ac.bd (S.Z.); salim.geb@ru.ac.bd (M.S.U.); 2 Bangladesh Reference Institute for Chemical Measurements, BRiCM, Bangladesh Council of Scientific and Industrial Research, Dhanmondi, Dhaka 1205, Bangladesh; mirolapharma31@gmail.com (M.A.); razu_ss86@yahoo.com (M.H.R.); malakhan_07@yahoo.com (M.K.); 3 Department of Genetic Engineering and Biotechnology, University of Rajshahi, Rajshahi 6205, Bangladesh; swagota33@gmail.com (S.B.R.G.); suvrobiswas0@gmail.com (S.B.); 4 Research Institute on Terrestrial Ecosystems (IRET)-UOS Naples, National Research Council of Italy (CNR), via P. Castellino 111, 80131 Naples, Italy; nunzio.cacciola@iret.cnr.it; 5 Department of Pharmacy, BGC Trust University Bangladesh, Chittagong 4381, Bangladesh; 6 Department of Agricultural Sciences, University of Naples Federico II, 80055 Portici, Italy; 7 Nutrition and Bromatology Group, Department of Analytical and Food Chemistry, Faculty of Food Science and Technology, Ourense Campus, University of Vigo, E32004 Ourense, Spain

**Keywords:** SARS-CoV-2, *Avicennia officinalis*, main protease, GC-MS, antioxidant, molecular dynamics simulation

## Abstract

The recent coronavirus disease 2019 (COVID-19) pandemic is a global threat for healthcare management and the economic system, and effective treatments against the pathogenic severe acute respiratory syndrome coronavirus 2 (SARS-CoV-2) virus responsible for this disease have not yet progressed beyond the developmental phases. As drug refinement and vaccine progression require enormously broad investments of time, alternative strategies are urgently needed. In this study, we examined phytochemicals extracted from *Avicennia officinalis* and evaluated their potential effects against the main protease of SARS-CoV-2. The antioxidant activities of *A. officinalis* leaf and fruit extracts at 150 µg/mL were 95.97% and 92.48%, respectively. Furthermore, both extracts displayed low cytotoxicity levels against *Artemia salina*. The gas chromatography–mass spectroscopy analysis confirmed the identifies of 75 phytochemicals from both extracts, and four potent compounds, triacontane, hexacosane, methyl linoleate, and methyl palminoleate, had binding free energy values of −6.75, −6.7, −6.3, and −6.3 Kcal/mol, respectively, in complexes with the SARS-CoV-2 main protease. The active residues Cys145, Met165, Glu166, Gln189, and Arg188 in the main protease formed non-bonded interactions with the screened compounds. The root-mean-square difference (RMSD), root-mean-square fluctuations (RMSF), radius of gyration (Rg), solvent-accessible surface area (SASA), and hydrogen bond data from a molecular dynamics simulation study confirmed the docked complexes′ binding rigidity in the atomistic simulated environment. However, this study′s findings require in vitro and in vivo validation to ensure the possible inhibitory effects and pharmacological efficacy of the identified compounds.

## 1. Introduction

The world is currently facing a challenging crisis caused by the novel severe acute respiratory syndrome coronavirus 2 (SARS-CoV-2), which causes coronavirus disease 2019 (COVID-19) and has introduced several unwanted changes to our lifestyles [1,2]. Although the virus reported in Wuhan City, China [1], it has spread worldwide, with reports of 2,799,924 deaths and 128,063,482 affected individuals as of 30 March 2021 (https://www.worldometers.info/coronavirus/, accessed on 12 March 2021). The World Health Organization (WHO) declared COVID-19 a global pandemic on 11 March 2020, and various preventative measures have been enacted, including shutting down schools and closing places where people gathered, such as bars, shopping malls, movie theaters, gymnasiums, and other sports venues [3,4]. The primary symptoms of COVID-19 are fever, cough, and pain, but they can be difficult to distinguish for the first 5 days of infection [3]. The angiotensin-converting enzyme 2 (ACE2) receptor serves as a prominent means through which SARS-CoV-2 gains entry to host cells. ACE2 is expressed not only in the respiratory system but also in a wide range of tissues, indicating that the viral attack can rapidly propagate throughout the body, augmenting the disease [4].

Both Middle East respiratory syndrome coronavirus (MERS-CoV) and SARS-CoV, similar to SARS-CoV-2, belong to the *Betacoronavirus* subfamily, sharing 82% [5] and 50% [6] similarity with SARS-CoV-2, respectively [7,8]. Although the fatality rate reported for SARS-CoV-2 (2.8%) is much lower than those for SARS-CoV (9.14%) and MERS-CoV (34.4%), SARS-CoV-2 is highly contagious, and the virus can spread via individuals who are infected but asymptomatic [9]. The SARS-CoV-2 viral fusion protein is surrounded via spike (S) proteins which interact with receptors on the host cell membrane, and the cleavage of the SARS-CoV-2 S protein at the S1 and S2 sites is required for viral penetration into the host cell [8]. The crystal structure of the SARS-CoV-2 main protease (M^pro^) was recently submitted to the Protein Data Bank (PDB) [7], revealing 11 putative sites of injection, consisting of type-I (Chymotrypsin), type-II (Picornavirus), and type-III domains. The type-I and type-II domains consist of six-stranded antiparallel β-barrels containing H-41 and C-145 active sites [10], whereas the type-III domain contains α-helices [11]. The SARS-CoV-2 life cycle depends on M^pro^ activity, and the absence of the M^pro^ protein in humans makes this protein a promising target for vaccine development [12,13,14].

Plants have been used as therapeutic agents since ancient times. Numerous studies have examined various plant extracts, and the secondary metabolites found in plants may have the potential to treat SARS-CoV-2 [15]. *Avicennia officinalis* is a popular evergreen medicinal mangrove plant found in Bangladesh, and this species can also be found in Europe, Western Asia, and North Africa [16]. Commonly known as Bain or Indian mangrove, *A. officinalis* has various medicinal effects, including antidiabetic, anti-inflammatory, anticancer, antioxidant, and antimicrobial properties [13]. Traditionally, the plant has been used to treat various maladies, such as asthma, dyspepsia, rheumatism, paralysis, and tumors. Moreover, *A. officinalis* is an influential plant due to its antioxidant properties [12,13,14], and natural antioxidant particles may act against viruses by denaturing the enzymes involves in viral replication. Therefore, *A. officinalis* may be useful in the fight against COVID-19 [17]. Importantly, this plant has previously exhibited significant in vitro anti-human immunodeficiency virus (HIV) attributes [18]. The phytochemicals in this plant might represent a potential source of compounds able to deactivate SARS-CoV-2 M^pro^.

The root extracts of *Baphia racemose* and *Sansevieria hyacinthoides* act against the Listeriosis disease (which is evolved by *Listeria monocytogenes*), as the extracts exhibit an absolute antibacterial effect against *Listeria monocytogenes* [19]. The leaf and twig extracts of *Archidendron clypearia* and seed extracts of *Washingtonia filifera* can inhibit the xanthine oxidase (XO), an enzyme that is liable for the development of gout disease and hyperuricemia [20,21]. Piperine, an ethanol extract compound of the fruits of *Piper longum* L. that suppress hemorrhage, and alleviates the lethality of Russell’s viper (*Doboia russelii*) venom in vivo [22,23]. Elderberry extract demonstrates a proficient effect against influenza A and B virus, whereas black soybean extract subdues coxsackievirus B1 and human adenovirus (type 1) [24,25,26]. The key antimicrobial compound of *Allium sativum* extract is allicin and the trypsin-like protease activity (which involves in developing periodontitis disease) of *Porphyromonas gingivalis* nearly entirely inhibited by allicin [27]. In vitro anti-plasmodial function in opposition to *Plasmodium falciparum* is exhibited by *Adenia cissampeloides, Terminalia ivorensis,* and *Elaeis guineensis* crude extracts [28]. Seed extracts of *Moringa oleifera* like 4-(4′-O-acetyl-a-L-rhamnopyranosyloxy) benzyl isothiocyanate and niazimicin act as a brawny suppressor of Epstein-Barr virus, which is a phorbol ester (TPA)-induced virus [29,30].

Computer-aided drug designing involves the generation of an artificial environment that can simulate the environment found in the human body [31], which can be used to examine the interactions between a target protein and bioactive compounds under simulated physiological conditions in a time-saving and cost-effective manner. In our investigation, both in vitro and in silico analyses were used to identify the top candidate phytochemicals derived from *A. officinalis* with the potential to inhibit SARS-CoV-2 M^pro^ active sites, and molecular dynamics studies were performed to predict the formation of probable ligand-receptor complexes with optimized conformations [32].

## 2. Results

### 2.1. DPPH Antioxidant Scavenging Assay

**Figure 1:**
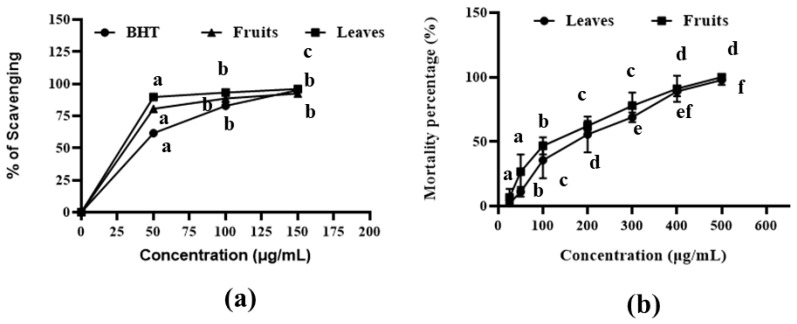
Antioxidant and cytotoxic activity of *A. officinalis* leaves and fruits extract. (**a**) DPPH scavenging activity of both leaves and fruits, and (**b**) mortality percentage of brine shrimp for cytotoxicity test where different significance letter indicates significant differences between mean ± SD of replication (n = 3) at a *p* < 0.05 significance level.

Both leaf and fruit extracts showed the highest DPPH free radical scavenging activities of 95.97% and 92.48% at the 150 µg/mL concentration. The half-maximal inhibitory concentration (IC_50_) values for the leaf and fruit extracts were 41.17 µg/mL and 47.22 µg/mL, respectively, whereas the IC_50_ value for BHT was 58.89 µg/mL (Figure 1a and Table S1). These results indicated that both extracts had moderately high antioxidant activities, with the leaf extract exhibiting better results than the fruit extract.

### 2.2. Cytotoxic Assay

The brine shrimp lethality results for the *A. officinalis* leaf and fruit methanolic extracts are shown in Figure 1b and Table S2. The leaf and fruit extracts showed cytotoxic properties, with LC_50_ values of 217.77 µg/mL and 179.78 µg/mL, respectively (Table S2). The results indicated a positive correlation between the concentrations of the extracts and the brine shrimp mortality rate.

### 2.3. GC-MS Analysis

**Figure 2:**
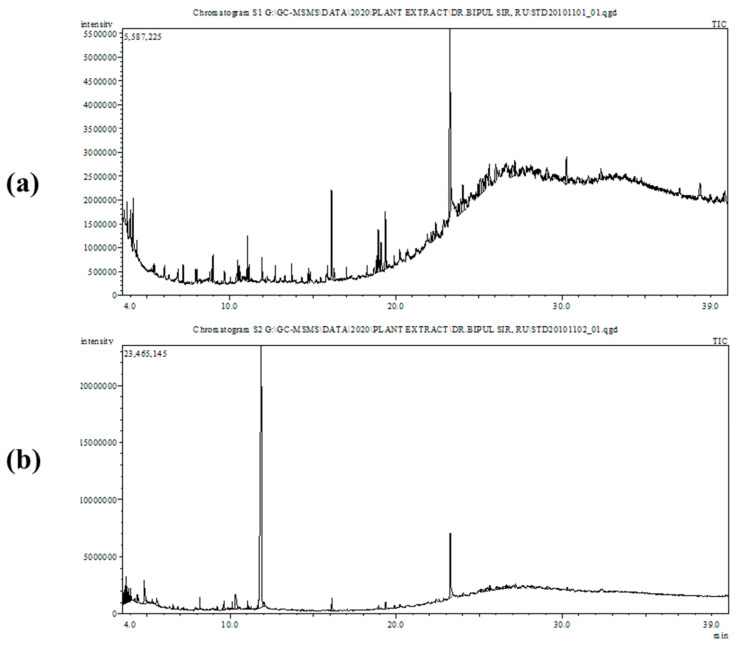
Total ionic chromatogram of *A. officinalis* methanolic extract of (**a**) leaves and (**b**) fruits by GC-MS.

The GC-MS analysis (Figure 2) identified 34 chemical compounds in the *A. officinalis* leaf extract and 41 chemical compounds in the fruit extract. The 3D structures of the identified compounds are shown in Figure S1. Most of the identified compounds have been previously associated with significant biological functions. The name, molecular formula, molecular mass, retention time, and percentage (%) area of each compound are denoted in Tables S3 and S4. The major compounds from the leaf extract included 9-octadecenamide (21.818%), hexadecenoic acid methyl ester (7.715%), methyl stearate (5.251%), and 9-octadecenoic acid methyl ester (4.330%). The most prominent compounds from the fruit extract were methyl alpha-d-galactopyranoside (55.24%), 9-octadecenamide (8.07%), and 1, 2-cyclopentanedione (3.39%).

### 2.4. Docking Analysis

**Figure 3:**
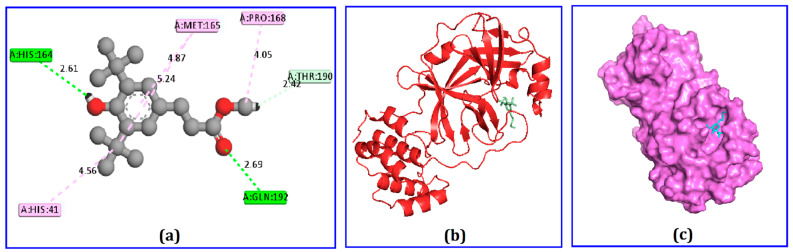
Binding interaction of the hydrocinnamic acid and main protease enzyme, (**a**) 2D representation of binding interaction, (**b**) 3D representation, and (**c**) surface view.

**Figure 4:**
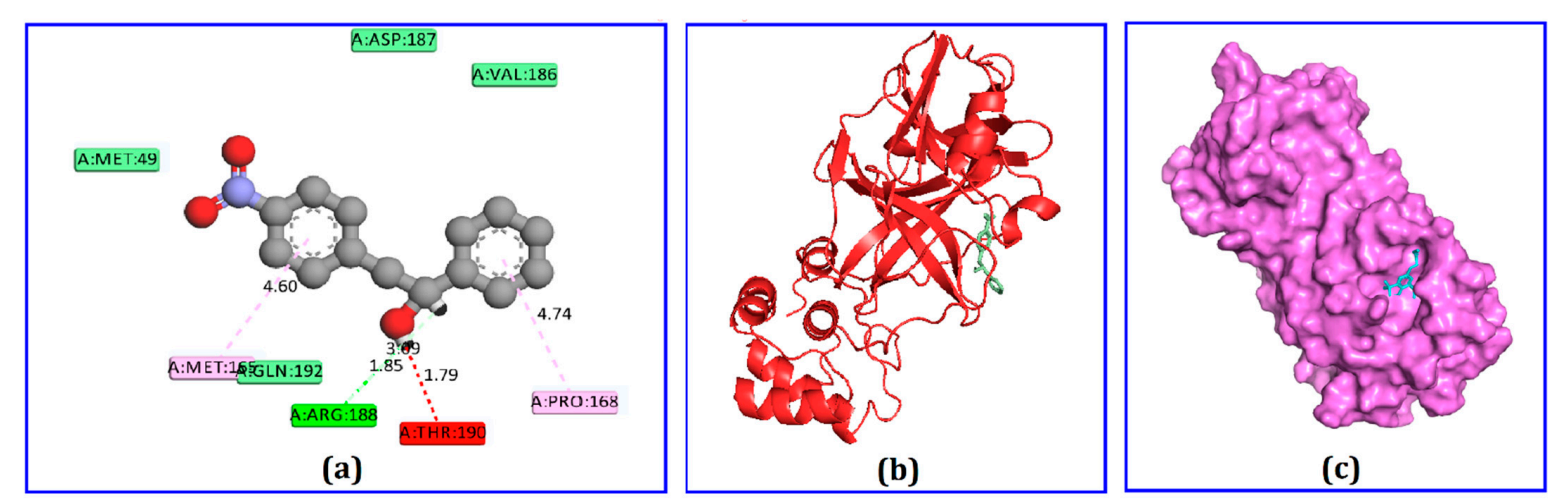
The non-bonded interaction of phenethyl alcohol and main protease from SARS-CoV-2. (**a**) 2D interaction pattern of main protease from SARS-CoV-2 and hexacosane, (**b**) 3D binding interaction, and (**c**) surface view of the docked complex.

**Figure 5:**
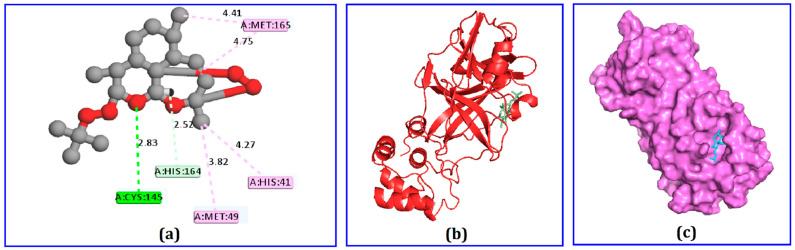
The interaction of dihydroartemisinin and main protease enzyme, (**a**) 2D interaction obtained from Discovery Studio, (**b**) 3D view, and (**c**) surface view.

Among the 75 identified phytochemicals, only three were selected for further analysis based on their low binding scores in the docking program. A computer-based approach, cognizant as AutoDock, was utilized to estimate the binding affinity of the plausible antiviral phytochemicals, which exhibited −7.5, −7.3, and −7.0 Kcal/mol binding affinity scores for hydrocinnamic acid, phenethyl alcohol, and dihydroartemisinin, respectively (Table 1). The compound hydrocinnamic acid created three hydrogen bonds with M^pro^ at His164, Gln192, and Thr190, two alkyl bonds at Pro168 and Met165, and one pi-alkyl bond at His41 (Figure 3 and Table 2). Phenethyl alcohol complexed with M^pro^ was stabilized by six hydrogen bonds at Arg188, Val186, Thr190, Gln192, and Met49 and two pi-alkyl bonds at Met165 and Pro168 (Figure 4 and Table 2). Dihydroartemisinin complexed with M^pro^ formed two hydrogen bonds at Cys145 and His164, two alkyl bonds at Met16 and Met49, and only one pi-alkyl bond at His41 (Figure 5 and Table 2).

### 2.5. Molecular Dynamics

**Figure 6:**
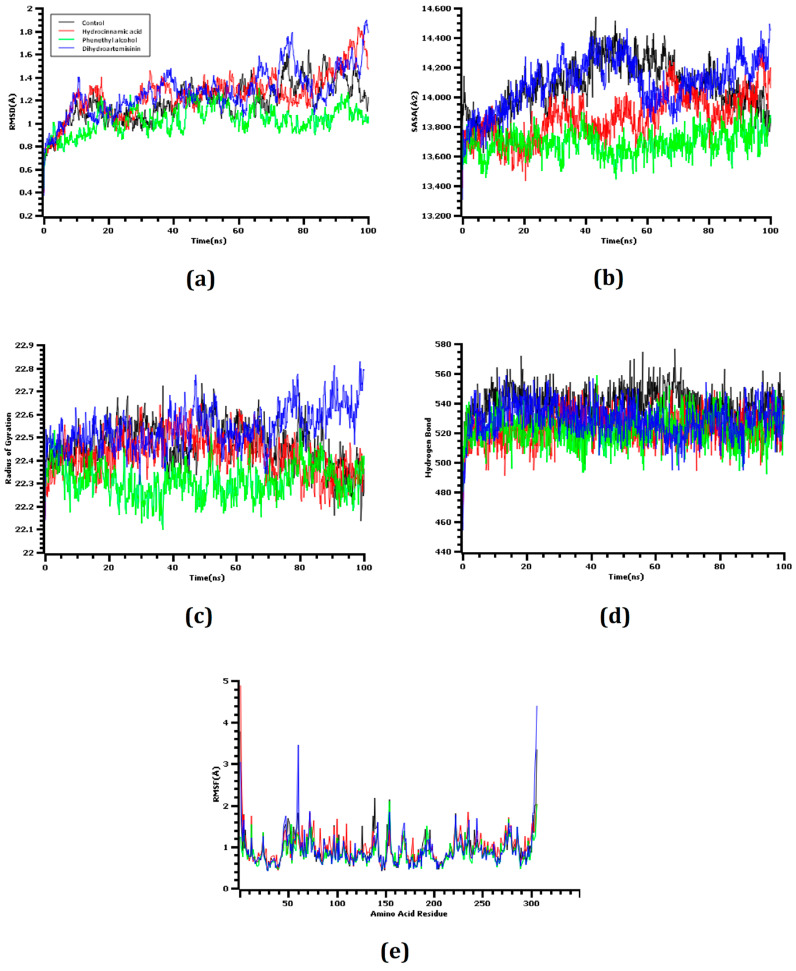
The molecular dynamics simulation. (**a**) Root mean square deviation of the control and four docked complexes, (**b**) solvent accessible surface area, (**c**) radius of gyration, (**d**) hydrogen bond of the docked and control complexes, and (**e**) root mean square fluctuation.

The RMSD values of the C-alpha atoms were determined from molecular dynamics simulation trajectories during observations of four docked complexes. The co-crystalized ligand was used as a control in this study. Figure 6 observed that the dihydroartemisinin had a higher degree of RMSD value than the control and other docked complexes. This result correlates with the higher flexibility of this complex. The other docked complexes were in between 1–2 Å, which demonstrates the high structural stability of the complexes.

Furthermore, the SASA values of the simulated complexes were analyzed to evaluate changes in protein surface exposure to the solvents. Higher SASA values indicate the expansion of the surface area, whereas lower SASA values indicate the compression of the protein volume. Figure 6 demonstrates that the complex between M^pro^ and dihydroartemisinin had a higher SASA value than all of the other evaluated docked complexes or the control system. The SASA profile of the M^pro^–dihydroartemisinin complex increased from the start of the simulation and had a similar SASA profile from 10 to 50 ns simulation time. Therefore, the control and dihydroartemisinin both resulted in a reduced SASA profile, which indicated the reduction of the protein surface area. Hydrocinnamic acid also resulted in a lower SASA value than the control complex, and the complex was observed to be flexible throughout the entire simulation. The other docked complex exhibited a lower degree of fluctuation compared with the control system, which indicated the stable nature of the docked complex.

The Rg value of a complex defines the compactness of the biological system. Both dihydroartemisinin and hydrocinnamic acid had similar Rg profiles and remained in a steady-state conformation. The other complex had a lower degree of deviation, which might be responsible for the protein′s compacted nature. The hydrogen bonds of the docked complexes were analyzed because hydrogen bonds play vital roles in maintaining protein integrity and stability. Dihydroartemisinin, hydrocinnamic acid, and phenethyl alcohol all formed a high number of hydrogen bonds across the simulation trajectory, suggesting that the ligand molecule bound tightly with SARS-CoV-2 M^pro^.

The RMSF values of the amino acid residues were analyzed to understand the flexibility across the complexes. Figure 6 demonstrates that almost every amino acid residue in all of the complexes had RMSF descriptor profiles below 2.5 Å. Low RMSF values are associated with a reduced degree of flexibility.

Final snapshots of the docked complexes were obtained at the end of the simulation trajectories to explore any changes in the binding residues (Table 3). Hydrocinnamic acid and M^pro^ formed three hydrogen bonds at Gln192 (active site), Gln189 (active site), and Ala191 (active site), and two alkyl bonds at Pro168 (active site) and Met165 (active site). Phenethyl alcohol and M^pro^ formed three hydrogen bonds at Glu166 (active site), Thr190 (active site), and Asp187, two pi-alkyl bonds at Leu167 and Pro168 (active site), and one alkyl bond at Met165 (active site). Dihydroartemisinin and M^pro^ formed two hydrogen bonds at His41 (active site) and Gln189 (active site), two alkyl bonds at Met165 (active site) and Leu167, and three pi-alkyl bonds at Pro168 (active site), Cys44, and Met49. From the literature, Glu166 can play an essential role in interacting with the main protease of SARS-CoV-2. Since the positioned at the active points of the targeted enzymes, it may interfere with the main protease function [33]. The previous study also suggests that His41 and Cys145 had multiple interactions with the main protease, which we also found for our screened compounds [34]. These results had consistency with previously published research works.

### 2.6. ADMET Analysis

The ADME profiles for ligand molecules were assessed to determine their drug-likeness properties. Drug-like compounds should have molecular weights below 500 Daltons, and all of our screened compounds had molecular weights below this cutoff value. The logP values of the screened molecules were found to be 4.09, 2.87, and 3.941, respectively (Table 4) for hydrocinnamic acid, phenethyl alcohol, and dihydroartemisinin. The surface areas for hydrocinnamic acid, phenethyl alcohol, and dihydroartemisinin were 127.28, 104.66, and 149.825 Å^2^, respectively (Table 4).

The ability to cross the blood–brain barrier and intestinal human absorption were found to be positive for all three ligand molecules. Toxicity assessments must be appropriately confirmed to ensure the safety of the tested ligand molecules. All three screened ligands were found to be non-carcinogenic according to hepatotoxicity and Ames toxicity profiling.

## 3. Discussion

A useful and cost-effective approach for extracting and purifying protein, and the COVID-19 pandemic, caused by SARS-CoV-2, has created the world’s most substantial medical crisis, associated with high mortality and rapid transmission. Although several studies are being performed in world-renowned laboratories worldwide, the development of treatment agents is time-dependent and subject to extensive experimental testing [35].

Plants have been used since ancient times to treat various diseases. According to the WHO, approximately 80% of the world’s population depends on the traditional plant-based medicinal systems [36]. The secondary metabolites found in plants, including alkaloids, flavonoids, organosulfur, terpenoids, limonoids, polyines, lignans, furyl compounds, thiophenes, proteins, peptides, polyphenolics, coumarins, and saponins, have been used as alternatives to synthetic particles for the development of drugs because many synthetic particles have adverse effects on human health. Phytochemicals represent potential treatment options because they display scavenging antioxidant, antiviral, antibacterial, and antifungal activities and may induce cancer prevention, enzyme stimulation, or other hormonal functions [37]. The COVID-19 outbreak has spurred several attempts to identify whether any active compounds found in plants could inhibit the spread of COVID-19. Combined with molecular docking and molecular dynamics simulation studies, medicinal chemistry can be used to identify potent candidate molecules that can effectively bind the active groove of a catalytic protein and facilitate target inhibition [38,39].

Mangrove plants are potential sources of phytochemicals (phenols, flavonoids, steroids, terpenoids, and tannins) with antimicrobial, anticancer, and antioxidant properties [40]. In the current work, a phytochemical analysis was performed to identify the compounds found in methanol extracts from the leaves and fruits of the mangrove plant *A. officinalis*. The qualitative and quantitative analysis [41] of *A. officinalis,* using GC-MS analysis, this plant to be an efficient source of more than 75 bioactive compounds (Figure 2) belonging to versatile groups, including terpenes, stearic acids, phenolics, alkaloids, alkenes, phenols, flavonoids, and fatty acids. Moreover, the presence of flavonoids and phenolic compounds is typically an indicator that a plant extract exerts antioxidant activities, and phytochemicals with antioxidant properties may be able to inhibit the enzymes that are essential for viral replication [13]. Our study indicated that both the leaf and fruit extracts displayed high antioxidant and cytotoxic activities (Figure 1). Previous findings have indicated that the presence of more polar metabolites, such as naphthoquinones and phenolics, can enhance the antioxidant activities of *A. officinalis* plant components [42]. A study also reported that this mangrove plant had a less toxic effect on Vero cell lines [43], which significantly supported our present investigation, in which the LC_50_ value determined by the brine shrimp lethality was less than 250 µg/mL [44].

Docking calculations can be used to identify potential candidate compounds from a diverse ligand library [42]. This study employed AutoDock Vina to predict the binding energies of the identified compounds based on the molecular docking study. The top binding scores exhibited by four compounds were selected from both docking programs. The top three candidate compounds, hydrocinnamic acid, phenethyl alcohol, and dihydroartemisinin, formed three, six, and two hydrogen bonds, respectively, when interacting with SARS-CoV-2 M^pro^. The high number of hydrogen bonds might be responsible for the better binding energy observed for these screened compounds [43]. Interestingly, these three candidate compounds were observed to form multiple hydrogen bonds with active sites of M^pro^, including Arg188, Arg187, Val186, Thr190, Gln192 Cys145, and His41 residues. Multiple bonds formed with the target receptor′s active site might inhibit and block the functions of these amino acids [45,46].

**Figure 7:**
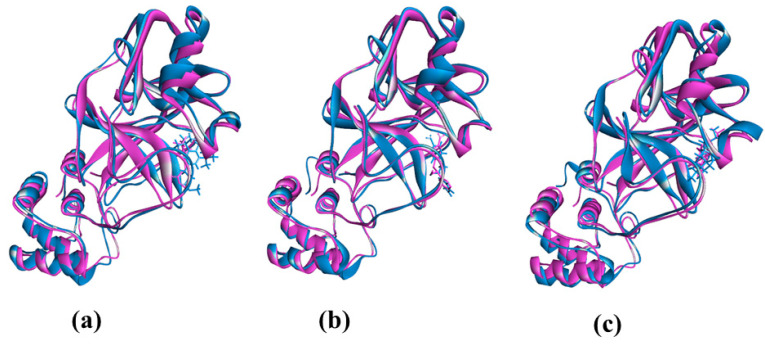
The superimposition between pre- and post-MD structure where lesser degrees of deviation were observed. The figures were prepared in the Pymol and Discovery Studio software (**a**–**c**).

**Figure 8:**
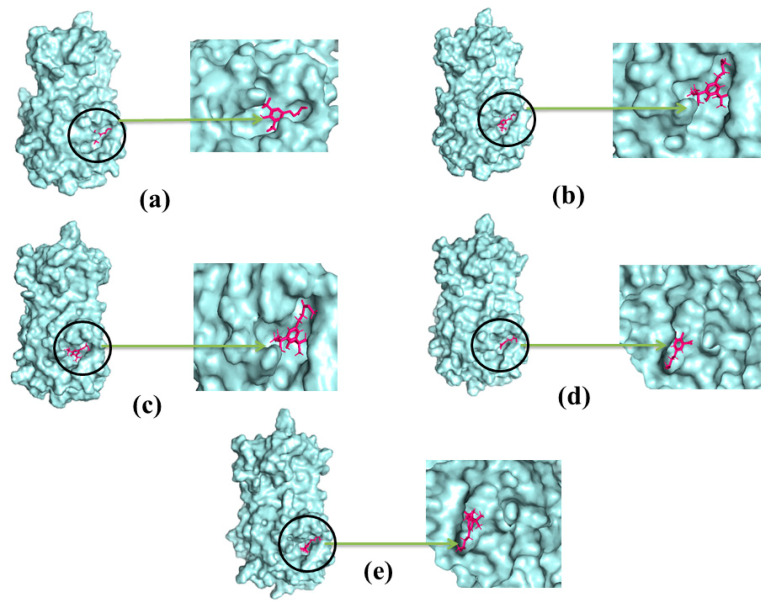
The simulation snapshots of hydrocinnamic acid and main protease complex acquired from the trajectories where rigid profiles of the ligand-protein complex were observed in the same binding pockets. The snapshots were (**a**–**e**) taken after 0, 25, 50, 75, and 100 ns intervals, respectively.

**Figure 9:**
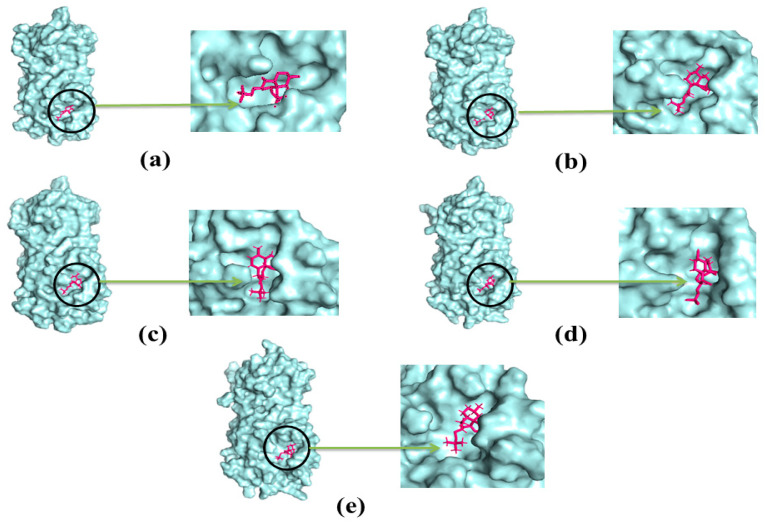
The dynamics snapshots (**a**–**e**) of phenethyl alcohol and main protease complex after 0, 25, 50, 75, and 100 ns, respectively.

**Figure 10:**
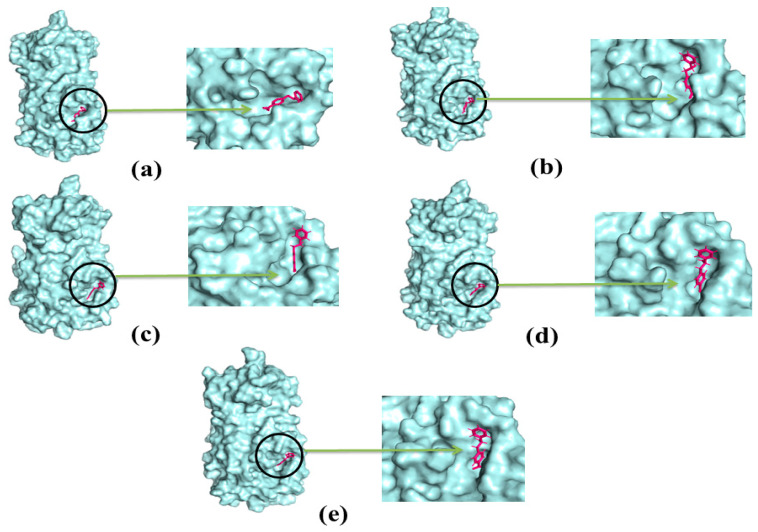
The simulation snapshots of dihydroartemisinin and main protease (**a**–**e**) after 0, 25, 50, 75, and 100 ns, respectively.

The molecular dynamics study was conducted to validate the docking study and to understand the dynamic motions and conformational changes that occur for the docked structure. Different descriptors from the simulation trajectories, including RMSD, RMSF, SASA, Rg, and hydrogen bonds, were assessed to confirm the structural integrity of the docked complexes. The RMSD and RMSF values of the docked complexes indicated that the complexes were stable under atomistic simulation conditions. The SASA profiles confirmed that the compounds experienced no changes in the surface area, except for dihydroartemisinin. The Rg and hydrogen bond values of the biological system revealed the stable nature of the complexes. The superimposition values of the pre- and post-molecular dynamics structures were 1.78 Å, 1.62 Å, and 1.92 Å, indicating few deviations in the docked structures (Figure 7). The simulation trajectories were analyzed, and snapshots from 25, 50, 75, and 100 ns were assessed, which showed no drastic changes in the binding pockets (Figure 8, Figure 9 and Figure 10).

The use of computational algorithms allows for the effective prediction of a compound’s drug-like properties, allowing for robust and cost-effective evaluations [47]. The molecular weights, surface areas, and LogP values of the screened compounds were found to be within the boundaries of Lipinski′s rule of five. Moreover, the ability to cross the blood–brain barrier and the human intestinal absorption rate [45] were found to be positive, indicating good absorption properties. Furthermore, the weak inhibition of hERG was found for all four hit compounds [46]. Thus, considering these circumstances, the methanolic *A. officinalis* fruit and leaf extracts could be used for therapeutic purposes in response to COVID-19.

## 4. Materials and Methods

### 4.1. Sample Collection

The leaves and fruits of *A. officinalis* were collected in July from the Dacope Upazila mangrove forest in Khulna, Bangladesh (located between 22°24′ and 22°40′ northern latitudes and between 89°24′ and 89°35′ east longitudes). Disease-free samples were placed in a sterile zip-top bag and transported to the Microbiology Laboratory, Department of Genetic Engineering and Biotechnology, University of Rajshahi, Rajshahi, Bangladesh. The collected materials were washed carefully under running tap water to remove dust and other contaminants. To remove the moisture contents, the samples were dried in an oven at 40 °C. The dried samples were finely powdered using an electric blender (Jaipan, Family mate, Mumbai, India) and stored in a plastic container at room temperature until further use.

### 4.2. Preparation of Plant Extract

The methanol extract was prepared according to the procedure described by Mariswamy et al. [48] by combining 25 g of the dry, ground, powdered samples with 150 mL of methanol solvent and left on a shaker for seven days. The extracts were then filtered through Whatman no. 1 filter paper to remove debris and dried using a hot air oven (LG oven, China) at a low temperature (less than 180 °C). The dried extracts were collected and stored at 40 °C for further analysis.

### 4.3. Antioxidant Activity

A DPPH (2,2-diphenyl-1-picryl-hydrazyl-hydrate; Sigma-Aldrich, Bengaluru, India) scavenging assay was used to analyze the antioxidant activity of our target compounds, based on the protocol described by Brand-Williams et al. [49]. In brief, 50, 100, and 150 µg/mL solid extracts derived from the leaves and fruits of *A. officinalis* were combined with DPPH (0.1 mM) at a ratio of 1:3, shaken, and incubated in the dark at room temperature for 30 min. The optical density (OD) was measured at 519 nm with an ultraviolet-visible (UV-Vis) spectrophotometer (Analytic Gena, Germany), using butylated hydroxytoluene (BHT; Sigma-Aldrich, India) as the standard [50]. The percentage of free radicals scavenged by each extract was calculated using the following equation.

$$\mathrm{Scavenging}\;\mathrm{activity}\;\left(\%\right)=\frac{\mathrm{AC}-\mathrm{AE}}{\mathrm{AC}}\times \;100$$

where AC = absorbance of the control and AE = absorbance of the plant extract.

### 4.4. Cytotoxicity Assay

An in vitro brine shrimp cytotoxic assay was performed to examine the toxicity of the plant extracts [51]. Briefly, *Artemia salina* (brine shrimp) was hatched in a specific tank at room temperature. The solvents were prepared at concentrations of 10, 100, and 1000 µg/mL were prepared. A total of 10 larvae were placed in each vial with 5 mL seawater and incubated for 24 h. Finally, a computer program was used to calculate the 50% lethal concentration (LC_50_).

### 4.5. Gas Chromatography-Mass Spectroscopy (GC-MS) Analysis

The bioactive compounds extracted from the leaves and fruits of *A. officinalis* were analyzed by gas chromatography-mass spectrometry (GC-MS) using the electron impact ionization (EI) method on a gas chromatograph (GC-MS, Shimadzu, Japan) coupled to a mass spectrometer (GC-MS TQ 8040, Shimadzu, Kyoto, Japan). A fused silica capillary column was used (Rxi-5 ms; 30 m, 0.25 mm ID, and 0.25 µm) with a column temperature set to 50 °C. The samples were injected in split mode by fixing the injection temperature at 250 °C. The oven temperature was set at 500 °C (for 1 min), then 200 °C (for 2 min), and finally held at 300 °C for 7 min. The names, structures, and molecular weights of the bioactive components in each extract were determined by matching their mass spectra with available data from the NIST and Wiley libraries [52,53]. GC-MS was performed for a total time of 39 min.

### 4.6. Ligand Preparation

The chemical compounds and their respective three-dimensional (3D) structure were retrieved from the PubChem database (www.pubchem.ncbi.nlm.nih.gov, accessed on 12 March 2021) in SDF format [53]. The chemical structures were initially optimized and prepared in Avogadro software [54] using the mmff94 force field with the steepest gradient approaches [55].

### 4.7. Protein Preparation

The SARS-CoV-2 M^pro^ structure was retrieved from PDB (www.rcsb.org, accessed on 12 March 2021) [56] with a resolution of 2.16 Å (PDB ID: 6LU7). The protein was initially cleaned, and water and heteroatoms were removed using Discovery Studio [57]. Later, the cleaned protein structure was minimized using YASARA software by AMBER14 force field [58].

### 4.8. Molecular Docking

A molecular docking study was performed to examine all 75 compounds identified in the *A. officinalis* fruit and leaf extracts using the AutoDock Vina [59] software package. The ligand was converted into an acceptable PDBQT format for AutoDock [60]. The box size and grid box center were set as (X:26.29 Å, Y:12.60 Å Z:58.94 Å) and (X:50.33 Å, Y:67.27 Å, Z:59.25 Å), respectively. Finally, the docking calculations were conducted in Pyrx (version 0.8), and binding interactions were analyzed in Discovery Studio (version 3.0) [61] and PyMol software (version 2.3) [62].

### 4.9. Molecular Dynamics Simulation

The molecular dynamics simulation study of the ligand–protein complexes were conducted in the YASARA dynamics software package (version 20.1.1) [63]. The AMBER14 force field [64] was used in this system, and protein–ligand complexes were initially cleaned and optimized. The initial energy minimization process was conducted by applying a simulated annealing method, in which the steepest gradient approaches were followed by 5000 cycles. The system was neutralized with the addition of water molecules around the protein complexes and 0.9% NaCl at 310 K [65]. The long-range electrostatic interactions were evaluated using the Particle Mesh Ewald method [66]. A cubic simulation cell was created, in which the cell size was maintained at 20 Å larger than all of the cases, and a periodic boundary condition was maintained. The time step was set to 1.25 fs, and after every 100 ps, the simulation trajectories were saved. The simulation trajectories were analyzed to calculate the root-mean-square deviation (RMSD), root-mean-square fluctuation (RMSF), the radius of gyration (Rg), solvent-accessible surface area (SASA), and hydrogen bonds [67].

### 4.10. ADMET (Absorption, Distribution, Metabolism, Toxicity) Analysis

The pharmacological properties of the ligands were assessed through ADMETSAR (http://lmmd.ecust.edu.cn/admetsar2/, accessed on 12 March 2021) [68,69] and PkCSM tools (http://biosig.unimelb.edu.au/pkcsm/, accessed on 12 March 2021) [70]. The canonical format of the chemical compounds was used as the entry system for ADMET (absorption, distribution, metabolism, toxicity) calculations.

### 4.11. Statistical Analysis

Data were analyzed by GraphPad Prism (version 8.4), and all the values are reported as the mean ± SEM (standard error of the mean). Values were evaluated as significantly different at *** *p* < 0.001, ** *p* < 0.01, and * *p* < 0.05, with one-way analysis of variance (ANOVA), followed by Dunnett’s test, whereas two-way ANOVA with repeated measures was used. The in vitro study was performed using triplicate measurements.

## 5. Conclusions

Plants have become a useful source of treatment due to their wide availability, reduced side effects, and increased specificity relative to synthetic compounds. Under pandemic conditions, in which no treatments are currently available, we assessed the ability of A. officinalis extracts to act as potent inhibitors of a viral target protein. The antioxidant and lethality assessments of the methanolic extracts showed significant effects, and the identified compounds were able to bind with the essential amino acid residues of SARS-CoV-2 M^pro^. Through molecular docking assays, hydrocinnamic acid, phenethyl alcohol, and dihydroartemisinin were identified as the three compounds with the highest targeted binding affinities for the active groove of SARS-CoV-2 M^pro^. The molecular dynamics simulation studies confirmed the structural rigidity of the docked complexes based on the analysis of several descriptors. This study may help future researchers identify an effective treatment for SARS-CoV-2 infections. Further experiments should explore the safety profiles of the candidate components derived from *A. officinalis*.

## Figures and Tables

**Table 1:** The docking energy score of top three ligand molecules from both extracts. The AutoDock software was used for cross docking validation.

Hit Compounds	Docking Scores (Kcal/mol)
Hydrocinnamic acid	−7.5
Phenethyl alcohol	−7.3
Dihydroartemisinin	−7.0

**Table 2:** The non-bonded interaction analysis from the top screened ligand molecules.

Compound	Amino Acid	Bond Type	Distance (Å)
Hydrocinnamic acid	His164 Gln192 Thr190 Pro168 Met165 His41	H H H A A PA	2.60 2.69 2.41 4.04 5.23 4.55
Phenethyl alcohol	Arg188 Arg187 Val186 Thr190 Gln192 Met49 Met165 Pro168	H H H H H H PA PA	2.18 2.01 2.76 2.52 2.33 2.48 4.47 4.58
Dihydroartemisinin	Cys145 His164 Met165 Met49 His41	H H A A PA	2.82 2.51 4.75 4.40 4.26

Here, H, A, PA, PS, PC indicates hydrogen, alkyl, pi-alkyl, pi-sulfur, pi-cation bond, respectively.

**Table 3:** Non-bonded interaction of the post-MD structures; here the binding residues were explored in Discovery Studio software package.

Complex	Residue	Bond Type	Distance (Å)
Hydrocinnamic acid	Gln192 Gln189 Ala191 Pro168 Met165	H H H A A	2.81 2.33 2.30 4.35 5.31
Phenethyl alcohol	Glu166 Thr190 Asp187 Leu167 Pro168 Met165	H H H PA PA A	1.87 2.59 2.92 4.89 4.25 4.52
Dihydroartemisinin	His41 Gln189 Met165 Leu167 Pro168 Cys44 Met49	H H A A PA PA PA	2.07 2.12 4.55 4.77 4.95 4.17 4.44

**Table 4:** The pharmacological assessment of the screened ligand molecules.

Properties	Hydrocinnamic Acid	Phenethyl Alcohol	Dihydroartemisinin
Molecular Weight	292.41	243.46	356.45
LogP	4.09	2.87	3.941
Surface Area	127.28	104.66	149.825
Blood Brain Barrier	0.8341 (+)	0.8805 (+)	0.9342 (+)
Human Intestinal Absorption	0.9346 (+)	0.9803 (+)	0.9352 (+)
P-Glycoprotein Inhibitor	0.8815 (-)	0.89978 (-)	0.8302 (-)
AMES Toxicity	0.8612 (-)	0.6173 (-)	0.6536 (-)
HERG Inhibition	0.9582 (WI)	0.6933 (WI)	0.9563 (WI)
Hepatotoxicity	No	No	No

Here, (+) indicates the positive result, (-) denotes negative, and WI indicates the weak inhibition. The molecular weight was calculated in Dalton, surface area Å^2^. The blood brain barrier, human intestinal absorption, P-glycoprotein inhibitors AMES toxicity, HERG inhibition, Hepatotoxicity were calculated on probability scale.

## Data Availability

Available data are presented in the manuscript.

## Supplementary Materials

The following are available online, Figure S1: Two dimensional (SD)-structure of *Avicennia officinalis* plant leaf compounds. Table S1: Free radical scavenging activity of methanolic extract of *Avicennia officinalis* with standard BHT concentrations, Table S2: Cytotoxic mortality percentage of leaf and fruits extract of *Avicennia officinalis* against *Artemia salina*, Table S3: Phytochemical compounds identification from *Avicennia officinalis* plant leaves extract by GC-MS, Table S4: Phytochemical compounds identification from *Avicennia officinalis* plant fruits extract by GC-MS. Table S5: Pharmacological assessment of all the compounds.

## Sample Availability

Samples of the compounds are available from the authors.
