# Supplementary material for: Efficacy of Phytochemicals Derived from Avicennia officinalis for the Management of COVID-19: A Combined In Silico and Biochemical Study
Source: Molecules. 2021 Apr 12;26(8):2210. doi: 10.3390/molecules26082210 (PMC8070553; doi:10.3390/molecules26082210)
Supplement: Supplementary file 1 [file molecules-26-02210-s001.pdf]

Supplementary Materials

Efficacy of Phytochemicals Derived from *Avicennia officinalis* for the Management of COVID-19: A Combined In Silico and Biochemical Study

Shafi Mahmud [1,†], Gobindo Kumar Paul [1,†], Mirola Afroze [2], Shirmin Islam [1], Swagota Briti Ray Gupt [3], Mamudul Hasan Razu [2], Suvro Biswas [3], Shahriar Zaman [1], Md. Salah Uddin [1], Mala Khan [2], Nunzio Antonio Cacciola [4], Talha Bin Emran [5,*], Md. Abu Saleh [1,*], Raffaele Capasso [6,*] and Jesus Simal-Gandara [7,*]

[1] Microbiology Laboratory, Department of Genetic Engineering and Biotechnology, University of Rajshahi, Rajshahi-6205, Bangladesh; shafimahmudfz@gmail.com (S.M.); gobindokumar38@gmail.com (G.K.P.); buligeb1127@gmail.com (S.I.); szaman@ru.ac.bd (S.Z.); salim.geb@ru.ac.bd (M.S.U.)

[2] Bangladesh Reference Institute for Chemical Measurements, BRiCM, Bangladesh Council of Scientific and Industrial Research, Dhanmondi, Dhaka-1205, Bangladesh; mirolapharma31@gmail.com (M.A.); razu_ss86@yahoo.com (M.H.R.); malakhan_07@yahoo.com (M.K.)

[3] Department of Genetic Engineering and Biotechnology, University of Rajshahi, Rajshahi-6205, Bangladesh; swagota33@gmail.com (S.B.R.G); suvrobiswas0@gmail.com (S.B.)

[4] Research Institute on Terrestrial Ecosystems (IRET)-UOS Naples, National Research Council of Italy, (CNR), via P. Castellino 111, 80131 Naples, Italy; nunzio.cacciola@iret.cnr.it (N.A.C.)

[5] Department of Pharmacy, BGC Trust University Bangladesh Chittagong 4381, Bangladesh

[6] Department of Agricultural Sciences, University of Naples Federico II, 80055 Portici, (Naples) Italy

[7] Nutrition and Bromatology Group, Department of Analytical and Food Chemistry, Faculty of Food Science and Technology, University of Vigo—Ourense Campus, E32004 Ourense, Spain

† These authors are contributed equally.

*Correspondence: jsimal@uvigo.es (J.S.-G.); rafcapas@unina.it (R.C); saleh@ru.ac.bd (M.A.S.); talhabmb@bgctub.ac.bd (T.B.E.)

Tel.: +34-988-387000 (J.S.-G.); +39-081678664 (R.C.); +88-01716-731747 (M.A.S.); +88-01819-942214 (T.B.E.)

Citation: Mahmud, S.; Paul, G.K.; Afroze, M.; Islam, S.; Gupt, S.B.R.; Razu, M.H.; Biswas, S.; Zaman, S.; Uddin, S.; Khan, M.; et al. Efficacy of Phytochemicals Derived from *Avicennia officinalis* for the Management of COVID-19: A Combined In Silico and Biochemical Study. *Molecules* **2021**, *26*, 2210. https://doi.org/10.3390/molecules26082210

Received: 12 March 2021
Accepted: 9 April 2021
Published: 12 April 2021

Publisher's Note: MDPI stays neutral with regard to jurisdictional claims in published maps and institutional affiliations.

Copyright: © 2021 by the authors. Submitted for possible open access publication under the terms and conditions of the Creative Commons Attribution (CC BY) license (http://creativecommons.org/licenses/by/4.0/).

Table S1. Free radical scavenging activity of methanolic extract of *Avicennia officinalis* with standard BHT concentrations.

Name of Samples	Conc. (μg/ml)	Absorbance (Mean ± SD)	% of Scavenging Activity	IC_{50} (μg/ml)
BHT	50	0.281 ± 0.007	61.56	11.24
	100	0.125 ± 0.059	82.85	
	150	0.036 ± 0.006	95.08	
Leaf	50	0.075 ± 0.010	89.73	41.17
	100	0.050 ± 0.007	93.17	
	150	0.029 ± 0.008	95.97	
Fruits	50	0.141 ± 0.021	80.56	47.22
	100	0.083 ± 0.010	88.54	
	150	0.055 ± 0.008	92.48	

Here, BHT: butylated hydroxytoluene.

Table S2. Cytotoxic mortality percentage of leaf and fruits extract of *Avicennia officinalis* against *Artemia salina.*

Concentration (μg/ml)	No. of Artemia Taken	Mean± SD value of Live Artemia		Mortality Percentage		LC_{50} μg/mL	
		Fruits	Leaf	Fruits	Leaf	Leaf	Fruits
25	15	14.00 ± 1.00	14.67 ± 0.58	6.67	2.22	217.77	179.78
50		11.00 ± 2.00	13.33 ± 0.58	26.67	11.11		
100		8.00 ± 1.00	9.67 ± 2.08	46.67	35.56		
200		5.67 ± 0.58	6.67 ± 2.08	62.22	55.56		
300		3.33 ± 1.53	4.67 ± 0.578	77.78	68.89		
400		1.33 ± 4.93	6.33 ± 0.578	91.11	88.89		
500		0.00 ± 0.00	0.33 ± 0.578	100	97.78		

Table S3. Phytochemical compounds identification from *Avicennia officinalis* plant leaves extract by GC-MS.

SL. No.	Compound Name	Retention Time	% Peak Area
1.	Name: (8Z,11Z,14Z)-icosa-8,11,14-trienoic acid<br>Formula:$C_{20}H_{34}O_2$<br>MW: 306.5	3.519	1.423
2.	Name: 2-(3methoxyanilino)benzoic acid<br>Formula: $C_{14}H_{13}NO_3$<br>MW: 243.26	3.796	1.675
3.	Name: 2,4-di-*tert*-butylphenol<br>Formula: $C_{14}H_{22}O$<br>MW: 206.32	11.05	2.656
4.	Name: Methyl (*Z*)-hexadec-9-enoate<br>Formula: $C_{17}H_{32}O_2$<br>MW: 268.4	15.775	1.160
5.	Name: Methyl hexadecanoate<br>Formula: $C_{17}H_{34}O_2$<br>MW: 270.5	16.127	7.715
6.	Name: Methyl 3-(3,5-di*tert*-butyl-4-hydroxyphenyl)propanoate<br>Formula: $C_{18}H_{28}O$<br>MW: 292.4	16.275	1.280
7.	Name: Methyl (9Z,12Z)-octadeca-9,12-dienoate<br>Formula: $C_{19}H_{34}O_2$<br>MW: 294.5	18.84	1.543

8.	Name: Methyl (*E*)-11-hydroperoxyoctadec-9-enoate<br>Formula: $C_{19}H_{36}O_4$<br>MW: 328.5	18.947	4.330
9.	Name: (*E*,7*R*,11*R*)-3,7,11,15-tetramethylhexadec-2-en-1-ol<br>Formula: $C_{20}H_{40}O$<br>MW: 296.5	19.117	3.254
10.	Name: Methyl octadecanoate<br>Formula: $C_{19}H_{38}O_2$<br>MW: 298.5	19.369	5.251
11.	Name: 1-iododotriacontane<br>Formula: $C_{32}H_{65}I$<br>MW: 576.8	20.615	1.160
12.	Name: (*Z*)-5-methylhenicos-6-en-11-one<br>Formula: $C_{22}H_{42}O$<br>MW: 322.6	22.135	1.208
13.	Name: 1,38-dibromooctatriacontane<br>Formula: $C_{38}H_{76}Br_2$<br>MW: 692.8	22.252	1.651
14.	Name: 1,54-dibromotetrapentacontane<br>Formula: $C_{54}H_{108}Br_2$<br>MW: 917.2	25.45	1.112

15.	Name: (*Z*)-*N*-(2-hydroxyethyl)octadec-9-enamide<br>Formula: $C_{20}H_{39}NO_2$<br>MW: 325.5	23.257	21.818
16.	Name: 1,54-dibromotetrapentacontane<br>Formula: $C_{54}H_{108}Br_2$<br>MW: 917.2	23.783	1.567
17.	Name: 2-methyltetracosane<br>Formula: $C_{25}H_{52}$<br>MW: 352.7	23.905	2.679
18.	Name: 2-methylhexacosane<br>Formula: $C_{27}H_{56}$<br>MW: 380.7	24.037	3.744
19.	Name: 6-(5-methylheptyl)oxan-2-one<br>Formula: $C_{13}H_{24}O_2$<br>MW: 212.33	25.03	1.423
20.	Name: 2-methylpropyl 2-methylpentanoate<br>Formula: $C_{10}H_{20}O_2$<br>MW:172.26	25.08	3.242
21.	Name: Heptadecane<br>Formula: $C_{41}H_{62}FN_5O_{10}$<br>MW: 804	25.239	1.842
22.	Name: Tetrapentacontane<br>Formula: $C_{54}H_{110}$<br>MW: 759.4	25.632	2.440

23.	Name: Dioctyl benzene-1,2-dicarboxylate<br>Formula: $C_{24}H_{38}O_4$<br>MW: 390.6	26.026	2.512
24.	Name: 1-bromotriacontane<br>Formula: $C_{30}H_{61}Br$<br>MW: 501.7	26.915	1.316
25.	Name: 3-octadecoxypropane-1,2-diol<br>Formula: $C_{21}H_{44}O_3$<br>MW:344.6	27.015	2.368
26.	Name: 2-hexyldecan-1-ol<br>Formula: $C_{17}H_{38}O_4S$<br>MW: 338.5	27.17	2.069
27.	Name: [(*E*)-non-1-enyl]cyclohexane<br>Formula: C_{15}H28<br>MW: 208.38	29.07	1.172
28.	Name: (*E*)-pentatriacont-17-ene<br>Formula: $C_{35}H_{70}$<br>MW: 490.9	29.091	1.220
29.	Name: 10-*tert*-butylperoxy-1,5,9-trimethyl-11,14,15,16-tetraoxatetracyclo[10.3.1.0^{4,13}.0^{8,13}]hexadecane<br>Formula: $C_{15}H_{24}O_2$<br>MW: 356.5	29.475	1.220
30.	Name: 2-hydroxy-4*a*,5-dimethyl-3-prop-1-en-2-yl-2,3,4,5,6,7,8,8*a*-octahydronaphthalen-1-one<br>Formula: $C_{15}H_{24}O_2$<br>MW: 236.35	30.1	1.280

31.	Name: (6*E*,10*E*,14*E*,18*E*)-2,6,10,15,19,23-hexamethyltetracosa-2,6,10,14,18,22-hexaene<br>Formula: $C_{30}H_{50}$<br>MW: 410.7	30.294	3.038
32.	Name: [(*E*)-2-(8-methoxy-2,15-dimethyl-14-pentacyclo[8.7.0.0^{2,7}.0^{5,7}.0^{11,15}]heptadecanyl)-6-methylhept-4-en-3-yl] 2,2-dimethylpropanoate<br>Formula: $C_{33}H_{54}O_{3}$<br>MW: 498.8	32.362	1.280
33.	Name: Ethane;propane;1-[(1E,3Z)-4-(4,4,5,5-tetramethyl-1,3,2-dioxaborolan-2-yl)cycloocta-1,3-dien-6-yn-1-yl]cyclopropane-1-carboxylic acid<br>Formula: $C_{25}H4_{3}BO_{4}$<br>MW: 418	38.315	1.950
34.	Name: 2-hydroxy-4*a*,5-dimethyl-3-prop-1-en-2-yl-2,3,4,5,6,7,8,8*a*-octahydronaphthalen-1-one<br>Formula: $C_{15}H_{24}0_{2}$<br>MW: 236.35	39.74	1.471

Table S4. Phytochemical compounds identification from *Avicennia officinalis* plant fruits extract by GC-MS.

SL. No.	Compound name	Retention Time	% Peak Area
1.	Name: 2,3,4,5,6,7,8-heptahydroxyoctanal<br>Formula: $C_8H_{16}O_8$<br>MW: 240.21	3.519	0.84
2.	Name: 1-propan-2-yl-3,6-diazatricyclo[4.3.1.1^{3,8}]undecan-9-one<br>Formula: $C_{12}H_{20}N_2O$<br>MW: 208.3	3.56	0.86
3.	Name: Butyl undec-10-enoate<br>Formula: $C_{15}H_{28}O_2$<br>MW: 240.38	3.59	0.88
4.	Name: 2,3-dimethoxy-1,4-dioxane<br>Formula: $C_6H_{12}O_4$<br>MW: 148.16	3.656	2.39
5.	Name: 3-ethoxypropane-1,2-diol<br>Formula: $C_5H_{12}O_3$<br>MW: 120.15	3.735	1.81
6.	Name: 3-[2-hydroxy-3-[(9Z,11Z)-octadeca-9,11-dienoyl]oxypropoxy]carbonylbenzoic acid<br>Formula: $C_3H_8O_3$<br>MW: 92.09	3.772	1.22
7.	Name: Cyclopent-2-en-1-one<br>Formula: C_5H_6O<br>MW: 82.1	3.829	1.00
8.	Name: 2-[2-(2-methoxyethoxy)ethoxy]ethyl 2-methylpropyl carbonate	3.915	0.55

	Formula: $C_{12}H_{24}O_6$<br>MW: 264.31		
9.	Name: (2*S*)-2-amino-3-methylbutan-1-ol<br>Formula: $C_5H_{13}NO$<br>MW: 103.16	4.407	1.02
10.	Name: Cyclopentane-1,2-dione<br>Formula: C_5H_6O2<br>MW: 98.1	4.817	3.39
11.	Name: Prop-2-enyl formate<br>Formula: $C_4H_6O_2$<br>MW: 86.09	5.575	1.23
12.	Name: 5-methyl-1*H*-pyrimidine-2,4-dione<br>Formula: $C_5H_6N_2O_2$<br>MW: 126.11	6.556	0.54
13.	Name: 2,4,6-trimethyloctane<br>Formula: $C_{11}H_{24}$<br>MW: 156.31	6.868	0.37
14.	Name: 2,3-dihydro-1-benzofuran<br>Formula: C_8H_8O<br>MW: 120.15	8.174	0.86
15.	Name: 4-ethenyl-2-methoxyphenol<br>Formula: $C_9H_{10}O_2$<br>MW: 150.17	9.227	0.38
16.	Name: Cyclohexylmethyl hexadecyl sulfite<br>Formula: $C_{23}H_{46}O_3S$<br>MW: 402.7	9.611	0.96

17.	Name: 6-(1-hydroxy-3-methylcyclohex-3-en-1-yl)-3-methylcyclohex-2-en-1-one<br>Formula: $C_{14}H_{20}O_2$<br>MW: 220.31	10.125	0.53
18.	Name: 2-(hydroxymethyl)-2-nitropropane-1,3-diol -<br>Formula: $C_4H_6NO_6P$<br>MW: 195.07	10.324	3.59
19.	Name: Methyl 3-hydroxypropanoate<br>Formula: $C_4H_8O_3$<br>MW: 104.1	10.465	0.42
20.	Name: 2,4-di*tert*-butylphenol<br>Formula: $C_{14}H_{22}O$<br>MW:206.32	11.049	0.53
21.	Name: 2-methylundecan-5-ol<br>Formula: C12$H_{26}O$<br>MW: 186.33	11.246	0.46
22.	Name: [(2*R*,3*R*,4*S*,5*R*,6*S*)-6-methoxy-3,4,5-tris(trimethylsilyloxy)oxan-2-yl]methanol<br>Formula: $C_{16}H_{38}O_6Si_3$<br>MW: 410.72	11.688	1.02
23.	Name: (2R,3S,4S,5R,6S)-2-(hydroxymethyl)-6-methoxyoxane-3,4,5-triol<br>Formula: $C_7H_{14}O_6$<br>MW: 194.18	11.852	55.24
24.	Name: Methyl hexadecanoate<br>Formula: $C_{17}H_{34}O_2$<br>MW: 270.5	16.121	1.21

25.	Name: Methyl (*Z*)-octadec-9-enoate<br>Formula: $C_{19}H_{36}O2$<br>MW: 296.5	18.947	0.39
26.	Name: Methyl octadecanoate<br>Formula: $C_{19}H_{36}O_{2}$<br>MW: 296.5	19.363	0.68
27.	Name: (*Z*)-*N*-(2-hydroxyethyl)octadec-9-enamide<br>Formula: $C_{38}H_{35}NO$<br>MW: 281.5	23.253	8.07
28.	Name: Ooctacosane<br>Formula: $C_{28}H_{58}$<br>MW: 394.8	24.037	0.40
29.	Name: Hexacontane<br>Formula: $C_{60}H_{122}$<br>MW: 843.6	25.092	0.35
30.	Name: 4-*O*-heptan-4-yl 1-*O*-tridec-2-ynyl butanedioate<br>Formula: $C_{24}H_{42}O_{4}$<br>MW:394.6	25.215	0.36
31.	Name: 11-methyltricosane<br>Formula: $C_{24}H50$<br>MW: 338.7	25.378	0.36
32.	Name: 1-nonoxyicosane<br>Formula: $C_{29}H_{60}O$<br>MW: 424.8	25.492	0.73
33.	Name: 1-(6-methylheptan-2-yl)-4-(4-methylpentyl)cyclohexane<br>Formula:$C_{20}H_{40}$	26.135	0.47

	MW: 280.5		
34.	Name: Undecan-5-ylcyclohexane<br>Formula: $C_{17}H_{34}$<br>MW: 238.5	26.248	0.35
35.	Name: Methyl 3-[methyl-(3-methyl-5-nitroimidazol-4-yl)amino]propanoate<br>Formula: $C_9H_{14}N_4O_4$<br>MW: 242.23	26.415	0.59
36.	Name: 1-propoxytetratriacontane<br>Formula: $C_{37}H_{76}O$<br>MW: 537	26.59	0.39
37.	Name: Tetratriacontyl 2,2,3,3,4,4,4-heptafluorobutanoate<br>Formula: $C_{38}H_{69}F_7O_2$<br>MW: 690.9	26.781	0.32
38.	Name: 11-methyltricosane<br>Formula: $C_{24}H_{50}$<br>MW: 338.7	27.178	0.33
39.	Name: 4-hexadecoxy-3-nitrobenzenesulfonyl fluoride<br>Formula: $C_{22}H_{36}FNO_2S$<br>MW: 445.6	28.145	0.52
40.	Name: 2-(3-ethyl-3,6,10,13,14-pentamethylpentadecyl)-1,1,3,6-tetramethylcyclohexane<br>Formula: $C_{32}H_{64}$<br>MW: 448.8	29.072	0.58
41.	Name: (6*E*,10*E*,14*E*,18*E*)-2,6,10,15,19,23-hexamethyltetracosa-2,6,10,14,18,22-hexaene<br>Formula: $C_{30}H_{50}$<br>MW:410.7	30.293	0.38

Table S5. Pharmacological assessment of all the compounds.

**Parameter**	**(8Z,11Z,14Z)-icosa-8,11,14-trienoic acid**	**2-(3methoxyanilino)benzoic acid**	**2,6-di*tert*-butyl-4-[(3,5-di*tert*-butyl-4-hydroxyphenyl)methyl]phenol**	**Methyl (*Z*)-hexadec-9-enoate**	**Methyl hexadecanoate**	**Methyl 3-(3,5-di*tert*-butyl-4-hydroxyphenyl) propanoate**	**Methyl (9*Z*,12*Z*)-octadeca-9,12-dienoate**	**Methyl (*E*)-8-hydroperoxyoctadec-9-enoate**
Molecular Weight	306.5	243.262	206.32	268.4	270.5	292.4	294.5	328.49
LogP	6.44	3.137	3.99	5.42	5.64	4.09	4.03	5.67
Surface Area	136.560	104.838	93.145	119.164	119.853	127.880	131.204	141.731
Blood Brain Barrier	0.9646 (+)	0.8603 (-)	0.9946 (+)	0.9958 (+)	1.0000 (+)	0.9102 (+)	0.9958 (+)	0.9675 (+)
Human Intestinal Absorption	0.9087 (+)	0.9149 (+)	0.9928 (+)	0.9567 (+)	0.6286 (-)	0.9918 (+)	0.9567 (+)	0.9419 (+)
P-Glycoprotein Inhibitor	0.6699 (-)	0.7873 (-)	0.9680 (-)	0.8083 (-)	0.8550 (-)	0.9272 (-)	0.7265 (-)	0.6029 (-)
AMES Toxicity	0.9674 (-)	0.8360 (-)	0.9494 (-)	0.9321 (-)	0.9765 (-)	0.8612 (-)	0.9296 (-)	0.8053 (-)
HERG Inhibition	0.9133 (WI)	0.9391 (WI)	0.9482 (WI)	0.8861 (WI)	0.9104 (WI)	0.9582 (WI)	0.9026 (WI)	0.8879 (WI)
Hepatotoxicity	No	No	No	No	No	No	No	No

Parameter	(*E*,7*R*,11*R*)-3,7,11,15-tetramethylhexadec-2-en-1-ol	Methyl octadecanoate	1-iododotriacontane	(*Z*)-5-methylhenicos-6-en-11-one	1,38-dibromooctatriacontane	1,54-dibromotetrapentacontane	(*Z*)-*N*-(2-hydroxyethyl)octadec-9-enamide	1,54-dibromotetrapentacontane
Molecular Weight	296.5	298.5	576.8	322.6	692.8	917.2	325.5	917.2
LogP		6.42	6.36[illegible] 14.14	7.64	15.82	22.06	5.13	22.06
Surface Area	133.778	132.583	225.314	145.875	271.977	373.816	143.489	373.816
Blood Brain Barrier	0.9375(+)	0.9848	1.000 (+)	0.9871 (+)	1.0000 (+)	1.0000(+)<br>1.0000(+)	0.9652 (+)	1.0000(+)<br>1.0000 (+)
Human Intestinal Absorption	0.9846(+)	0.9881 (+)	0.866 (+)	0.9797 (+)	0.9841 (+)	0.7509 (+)	0.8112 (+)	0.7509 (+)
P-Glycoprotein Inhibitor	0.8620 (-)	0.8222 (-)	0.7973 (-)	0.6918 (-)	0.7558 (-)	0.7558 (-)	0.7725 (-)	0.7558 (-)
AMES Toxicity	0.9132 (-)	0.9765 (-)	0.9550 (-)	0.9383 (-)	0.8988 (-)	0.8988 (-)	0.8778 (-)	0.8988 (-)
HERG Inhibition	0.7838 (WI)	0.9104 (WI)	0.8538 (WI)	0.7441 (WI)	0.7740 (WI)	0.7740 (WI)	0.9499 (WI)	0.7740 (WI)
Hepatotoxicity	No	No	No	No	No	No	No	No

Parameter	2-methyltetracosane	2-methylhexacosane	10-methyldodecan-5-olide	Heptadecane	Tetrapentacontane	Dioctyl benzene-1,2-dicarboxylate	1-bromotriacontane	3-octadecoxypropane-1,2-diol
Molecular Weight	352.7	3	172.26	240.475	759.4	390.6	501.7	344.6
LogP	9.85	10.63	2.62	6.88	21.31	5.67	12.32	6.94
Surface Area	161.498	174.228	75.299	110.578	346.081	170.550	207.190	150.740
Blood Brain Barrier	1.0000 (+)	1.0000 (+)	1.0000 (+)	1.0000 (+)	0.9821 (+)	0.9812 (+)	1.0000 (+)	0.9107 (+)
Human Intestinal Absorption	0.9588 (+)	0.9588 (+)	0.9838 (+)	0.8865 (+)	0.8865 (+)	1.0000 (+)	0.8662 (+)	0.8825 (+)
P-Glycoprotein Inhibitor	0.8321 (-)	0.7988 (-)	0.9603 (-)	0.9285 (-)	0.7233 (-)	0.6940 (+)	0.8219 (-)	0.8489 (-)
AMES Toxicity	0.9916 (-)	0.9916 (-)	0.9475 (-)	0.9965 (-)	0.9965 (-)	0.9504 (-)	0.9133 (-)	0.8907 (-)
HERG Inhibition	0.8929 (WI)	0.8929 (WI)	0.8033 (WI)	0.8620 (WI)	0.8620 (WI)	0.8980 (WI)	0.8800 (WI)	0.8696 (WI)
Hepatotoxicity	No	No	No	No	No	No	No	No

Parameter	2-hexyldecan-1-ol	1-cyclohexylnonene	[(*E*)-non-1-enyl]cyclohexane	10-*tert*-butylperoxy-1,5,9-trimethyl-11,14,15,16-tetraoxatetracyclo[10.3.1.0^{4,13}.0^{8,13}]hexadecane	(2-hydroxy-4*a*,5-dimethyl-3-prop-1-en-2-yl-2,3,4,5,6,7,8,8*a*-octahydronaphthalen-1-one	(6*E*,10*E*,14*E*,18*E*)-2,6,10,15,19,23-hexamethyltetracosa-2,6,10,14,18,22-hexaene	[(*E*)-2-(8-methoxy-2,15-dimethyl-14-pentacycloheptadecanyl)-6-methylhept-4-en-3-yl] 2,2-dimethylpropanoate	Ethane;propane;1-[(1E,3Z)-4-(4,4,5,5-tetramethyl-1,3,2-dioxaborolan-2-yl)cycloocta-1,3-dien-6-yn-1-yl]cyclopropane-1-carboxylic acid
Molecular Weight	338.5	208.38	490.9	356.5	236.35	410.7	498.8	418.4
LogP	4.82	5.48	13.6755	3.9411	2.9549	2.9549	8.08	4.6807
Surface Area	138.007	96.153	224.458	149.825	104.103	189.185	221.087	185.193
Blood Brain Barrier	0.9723 (+)	1.0000 (+)	1.0000 (+)	0.9642 (+)	0.8256 (+)	0.9962 (+)	0.8729 (+)	0.9746 (+)
Human Intestinal Absorption	0.8484 (+)	0.9603 (+)	0.9148 (+)	0.8171 (+)	0.9918 (+)	0.9975 (+)	0.9856 (+)	0.9610 (+)
P-Glycoprotein Inhibitor	0.8356 (-)	0.9578 (-)	0.5954 (-)	0.7059 (-)	0.9046 (-)	0.9803 (-)	0.7129 (-)	0.9046 (-)
AMES Toxicity	0.9798 (-)	0.9911 (-)	0.9901 (-)	0.6536 (-)	0.9457 (-)	0.9518 (-)	0.8666 (-)	0.5294 (-)
HERG Inhibition	0.8899 (WI)	0.7461 (WI)	0.8273 (WI)	0.9563 (WI)	0.9314 (WI)	0.7689 (WI)	0.9653 (WI)	0.9833 (WI)
Hepatotoxicity	No	No	No	No	No	No	No	No

Parameter	2-hydroxy-4*a*,5-dimethyl-3-prop-1-en-2-yl-2,3,4,5,6,7,8,8*a*-octahydronaphthalen-1-one	2,3,4,5,6,7,8-heptahydroxyoctanal	1-propan-2-yl-3,6-diazatricyclo[4.3.1.1^{3,8}]undecan-9-one	Butyl undec-10-enoate	2,3-dimethoxy-1,4-dioxane	3-ethoxypropane-1,2-diol	3-[2-hydroxy-3-[(9*Z*,11*Z*)-octadeca-9,11-dienoyl]oxypropoxy]carbonylbenzoic acid	Cyclopent-2-en-1-one
Molecular Weight	236.35	240.21	208.3	240.38	148.16	120.15	92.09	82.102
LogP	2.95	-4.66	0.46			[illegible]	[illegible]	0.9055
Surface Area	104.103	91.015	91.417	106.434	60.012	48.901	35.852	36.665
Blood Brain Barrier	0.8256 (+)	0.9028 (-)	0.9891 (+)	0.9974 (+)	0.9652 (+)	0.8485 (+)	0.6136 (+)	0.9845 (+)
Human Intestinal Absorption	0.9975 (+)	0.6807 (+)	0.9712 (+)	0.9962 (+)	0.8520 (+)	0.9907 (+)	0.923 (+)	0.9955 (+)
P-Glycoprotein Inhibitor	0.9046 (-)	0.9576 (-)	0.9575 (-)	0.8219(-)	0.9608 (-)	0.9855 (-)	0.9186 (-)-	0.8672 (-)
AMES Toxicity	0.9457 (-)	0.9132 (-)	0.7157 (-)	0.9406 (-)	0.6787 (-)	0.5335 (-)	0.8278 (-)	0.793 (-)
HERG Inhibition	0.9314 (WI)	0.9883 (WI)	0.8263(WI)	0.8776 (WI)	0.9400 (WI)	0.9403 (WI)	0.9670 (WI)	0.8463 (WI)
Hepatotoxicity	No	No	No	No	No	No	No	No

Parameter	2-[2-(2-methoxyethoxy) ethoxy]ethyl 2-methylpropyl carbonate	(2*S*)-2-amino-3-methylbutan-1-ol	Cyclopentane-1,2-dione	Prop-2-enyl formate	5-methyl-1*H*-pyrimidine-2,4-dione	2,4,6-trimethyloctane -	2,3-dihydro-1-benzofuran	4-ethenyl-2-methoxyphenol
Molecular Weight	264.31	103.16	98.1	86.09	126.11	156.31	120.15	150.17
LogP	1.4752	-5.0368	0.3085	0.3454	-0.62838	4.1048	1.6215	2.0438
Surface Area	108.483	103.779	41.516	36.420	50.559	72.389	54.269	65.744
Blood Brain Barrier	0.9353 (+)	0.7320 (-)	0.9819 (+)	0.9813 (+)	0.9790 (+)	0.9813 (+)	0.9868 (+)	0.8480 (+)
Human Intestinal Absorption	0.9887 (+)	0.7897 (-)	0.9898 (+)	0.9800 (+)	0.9742 (+)	0.9958 (+)	1.0000 (+)	0.9904 (+)
P-Glycoprotein Inhibitor	0.7541 (-)	0.5649 (-)	0.6700 (-)	0.9252 (-)	0.9303 (-)	0.8553 (-)	0.9353 (-)	0.6833 (-)
AMES Toxicity	0.6645 (-)	0.8389 (-)	0.5085 (+)	0.9133 (-)	0.9230 (-)	0.9947 (-)	0.6217 (-)	0.9132 (-)
HERG Inhibition	0.8835 (WI)	0.9936 (WI)	0.8807 (WI)	0.9604 (WI)	0.9562	0.9587	0.7106	0.8946
Hepatotoxicity	No	No	No	No	No	No	No	No

Parameter	Cyclohexylmethyl hexadecyl sulfite	6-(1-hydroxy-3-methylcyclohex-3-en-1-yl)-3-methylcyclohex-2-en-1-one	2-(hydroxymethyl)-2-nitropropane-1,3-diol -	Methyl 3-hydroxypropanoate	2,4-di*tert*-butylphenol	2-methylundecan-5-ol	[(2*R*,3*R*,4*S*,5*R*,6*S*)-6-methoxy-3,4,5-tris(trimethylsilyloxy)oxan-2-yl]methanol	(2R,3S,4S,5R,6S)-2-(hydroxymethyl)-6-methoxyoxane-3,4,5-triol
Molecular Weight	402.7	220.31	151.118	104.105	206.329	410.72	410.732	194.18
LogP	7.6599	2.7731	-2.0212	-0.4582	3.9872	3.0103	3.0103	-2.5673
Surface Area	171.781	97.048	56.870	41.903	93.145	156.082	156.082	75.327
Blood Brain Barrier	0.9564 (+)	0.9381(+)	0.5544 (+)	0.9660 (+)	0.9502(+)	0.8362 (+)	0.9868 (+)	0.6148 (-)
Human Intestinal Absorption	1.0000 (+)	1.0000 (+)	0.8455 (+)	0.9432 (+)	0.9941(+)	0.9404 (-)	1.0000 (+)	0.8373(-)
P-Glycoprotein Inhibitor	0.5805 (-)	0.5938 (-)	0.9072 (-)	0.9140 (-)	0.9160 (-)	0.5722 (-)	0.9353 (-)	0.8601 (-)
AMES Toxicity	0.5341 (-)	0.8317 (-)	0.8871 (-)	0.9320 (-)	0.9494 (-)	0.5613 (-)	0.6217 (-)	0.6078 (-)
HERG Inhibition	0.7276 (SI)	0.9297 (WI)	0.9200 (WI)	0.9641 (WI)	0.9482 (WI)	0.9463 (WI)	0.7106 (WI)	0.9535 (WI)
Hepatotoxicity	No	No	No	No	No	No	No	No

Parameter	Methyl hexadecanoate	Methyl (*Z*)-octadec-9-enoate	Methyl octadecanoate	(*Z*)-*N*-(2-hydroxyethyl) octadec-9-enamide	Octacosane	4-*O*-heptan-4-yl 1-*O*-tridec-2-ynyl butanedioate	Succinic acid, tridec-2-yn-1-yl 4-heptyl ester	11-methyltricosane
Molecular Weight	270.5	296.5	298.511	325.537	394.772	843.6	394.6	338.7
LogP	5.6407	6.1969	6.4209	5.1324	11.1688	23.652	6.356	9.4643
Surface Area	119.853	131.894	132.583	5.1324	180.593	384.271	172.682	155.133
Blood Brain Barrier	0.9848 (+)	0.9838 (+)	0.9848 (+)	0.9089 (+)	0.9821 (+)	0.9821 (+)	0.9578 (+)	0.9821 (-)
Human Intestinal Absorption	0.9881 (+)	0.9941 (+)	0.9881 (+)	0.9934 (+)	0.9921 (+)	0.9921 (+)	0.9310 (+)	0.9921 (-)
P-Glycoprotein Inhibitor	0.8951 (-)	0.8472 (-)	0.8951 (-)	0.8468 (-)	0.8985 (-)	0.8985(-)	0.7063 (-)	0.8985 (-)
AMES Toxicity	0.9765 (-)	0.9321 (-)	0.9765 (-)	0.8778 (-)	0.9965 (-)	0.9965(-)	0.9348 (-)	0.8109 (-)
HERG Inhibition	0.9104 (SI)	0.8861 (WI)	0.9104 (WI)	0.9499 (WI)	0.8620 (WI)	0.8620 (WI)	0.9324 (WI)	0.8620 (WI)
Hepatotoxicity	No	No	No	No	No	No	No	No

Parameter	1-nonoxyicosane	1-(6-methylheptan-2-yl)-4-(4-methylpentyl)cyclohexane	Undecan-5-ylcyclohexane	Methyl 3-[methyl-(3-methyl-5-nitroimidazol-4-yl)amino]propanoate	1-propoxytetratriacontane	Tetratriacontyl 2,2,3,3,4,4,4-heptafluorobutanoate	11-methyltricosane	4-hexadecoxy-3-nitrobenzene sulfonyl fluoride
Molecular Weight	424.8	280.5	238.5	242.23	537	690.9	338.7	445.6
LogP	10.7953	7.0815	6.3435	0.3276	13.9161	14.8656	9.4643	7.1131
Surface Area	192.071	128.667	109.572	97.948	242.991	282.676	155.133	180.123
Blood Brain Barrier	0.9870 (+)	0.9791 (+)	0.9825 (+)	0.8499 (+)	0.9870 (+)	0.9931 (+)	0.9813 (+)	0.8613 (+)
Human Intestinal Absorption	0.9958 (+)	0.9901 (+)	0.9950 (+)	0.9244 (+)	0.9958 (+)	1.0000 (+)	0.9914 (+)	1.0000 (+)
P-Glycoprotein Inhibitor	0.8414 (-)	0.9089 (-)	0.9100 (-)	0.6772 (-)	0.8414 (-)	0.8719 (-)	0.8729 (-)	0.6686(+)
AMES Toxicity	0.9102 (-)	0.9559 (-)	0.9957 (-)	0.7878 (-)	0.9102 (-)	0.9296 (-)	0.9934 (-)	0.5111(+)
HERG Inhibition	0.6690 (SI)	0.8517 (WI)	0.8373 (WI)	0.6104 (WI)	0.6690 (WI)	0.9546 (WI)	0.8522 (WI)	0.6502 (WI)
Hepatotoxicity	No	No	No	No	No	No	No	No

Parameter	2-(3-ethyl-3,6,10,13,14-pentamethylpentadecyl)-1,1,3,6-tetramethylcyclohexane	(6*E*,10*E*,14*E*,18*E*)-2,6,10,15,19,23-hexamethyltetracosa-2,6,10,14,18,22-hexaene
Molecular Weight	448.8	410.7
LogP	11.1863	10.605
Surface Area	205.047	189.185
Blood Brain Barrier	0.9876 (+)	0.9442 (+)
Human Intestinal Absorption	0.9956 (+)	0.9895 (+)
P-Glycoprotein Inhibitor	0.7808 (-)	0.7230 (-)
AMES Toxicity	0.9778 (-)	0.9518 (-)
HERG Inhibition	0.9177 (WI)	0.7689 (WI)
Hepatotoxicity	No	No

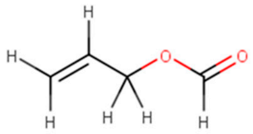

CID-61278

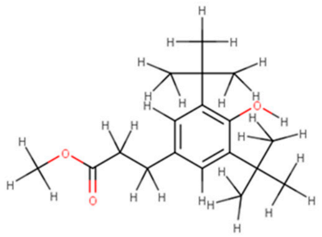

CID-62603

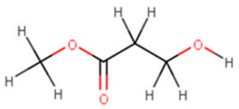

CID-80252

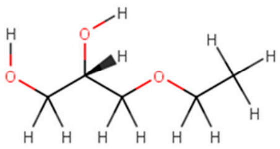

CID-94215

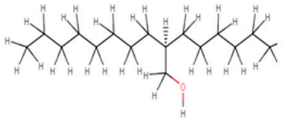

CID-95337

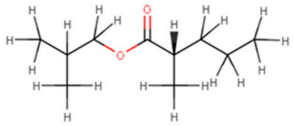

CID-226902

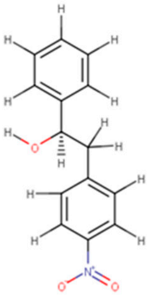

CID-285015

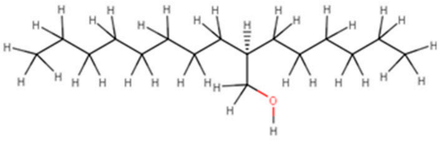

CID-524419

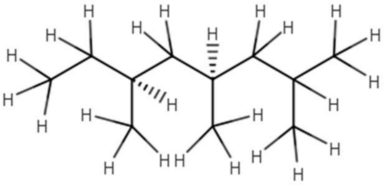

CID-545612

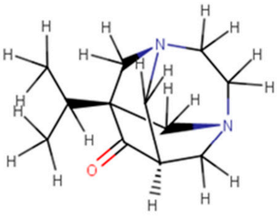

CID-547555

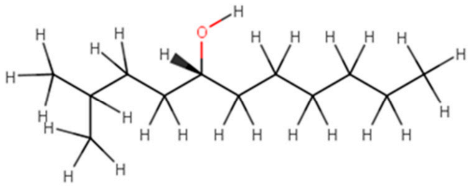

CID-544080

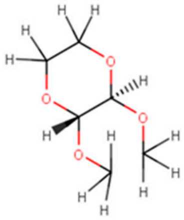

CID-548346

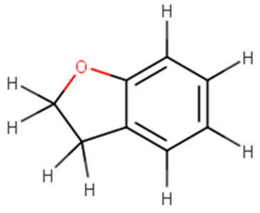

CID-10329

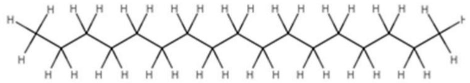

CID-12398

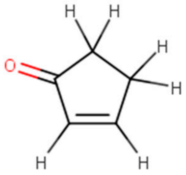

CID-13588

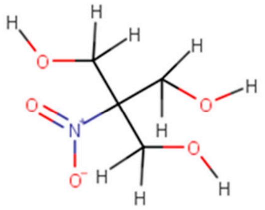

CID-31337

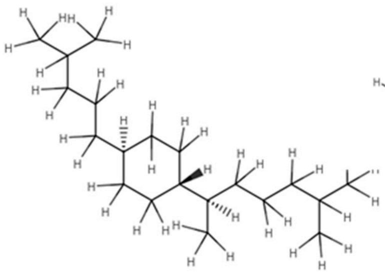

CID-41687

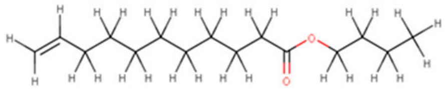

CID_61027

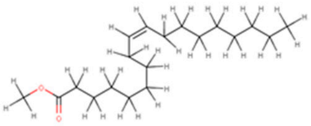

CID-5364509

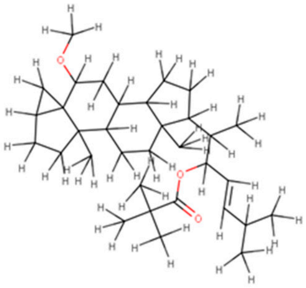

CID-5365019

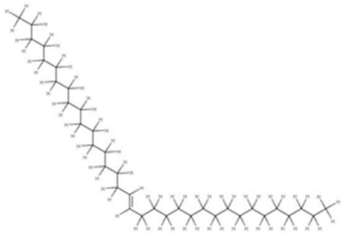

CID-5365022

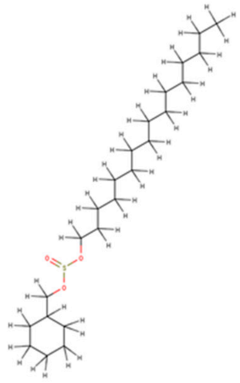

CID-6421705

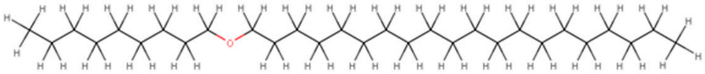

CID-87077398

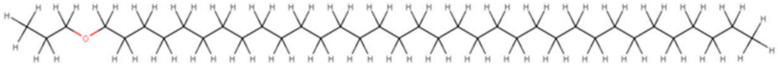

CID-91691504

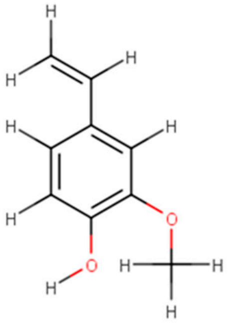

CID-332

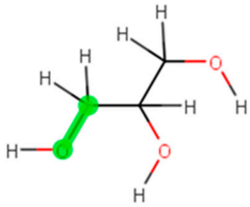

CID-753

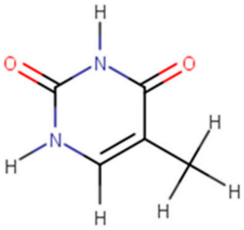

CID-1135

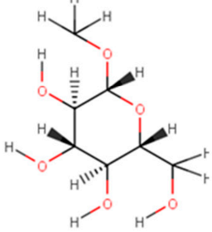

CID-2108

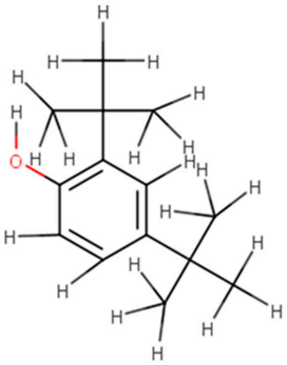

CID-7311

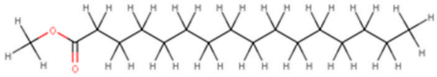

CID-8181

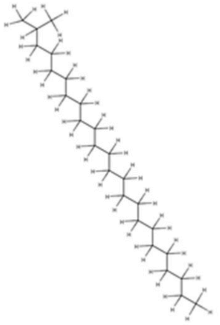

CID-527459

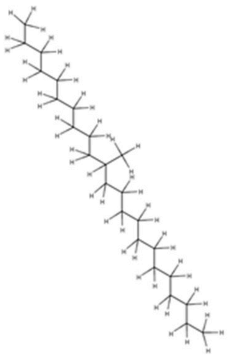

CID-530326

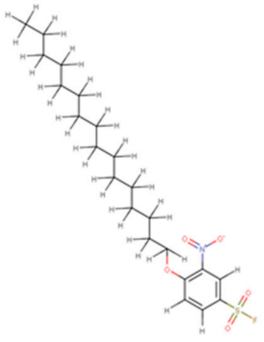

CID-5130516

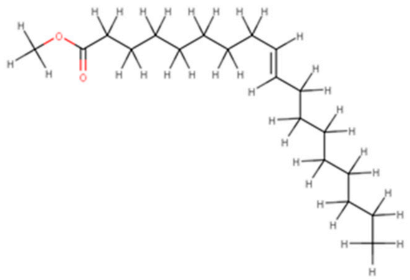

CID-5280590

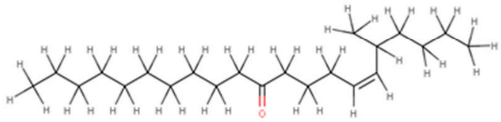

CID-5363254

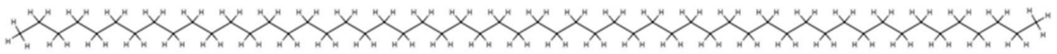

CID-521846

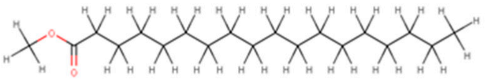

CID-8201

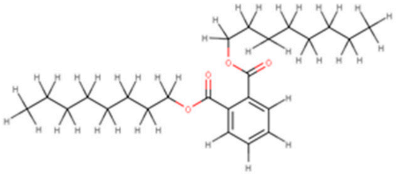

CID-8346

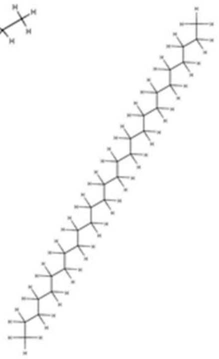

CID-12408

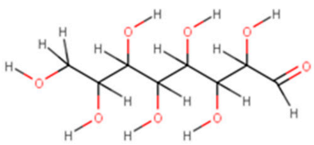

CID-521082

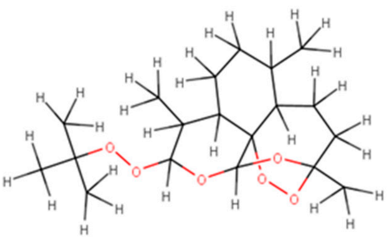

CID-537898

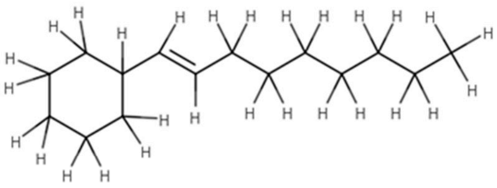

CID-5364533

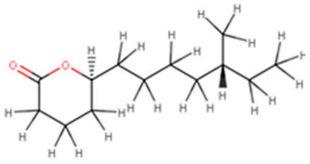

CID-21778196

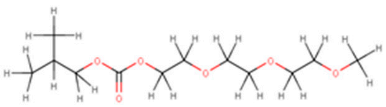

CID-91699246

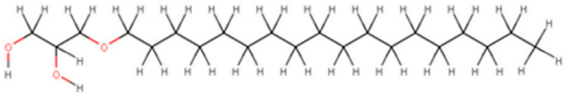

CID-3681

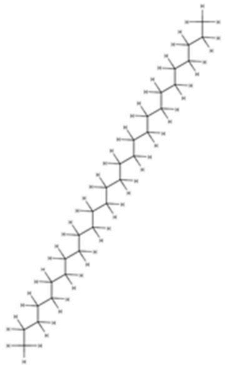

CID-24318

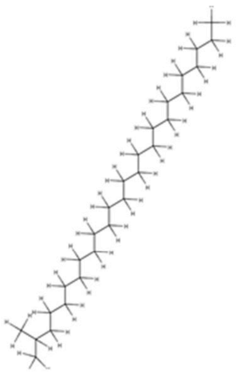

CID-150931

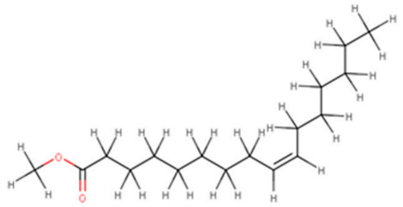

CID-643801

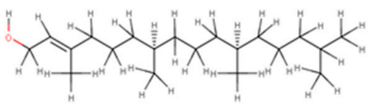

CID-5280435

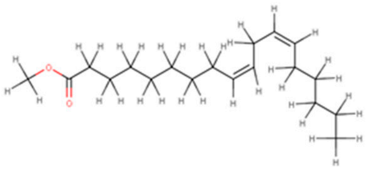

CID-5284421

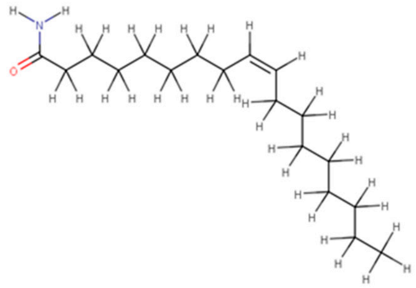

CID-5283387

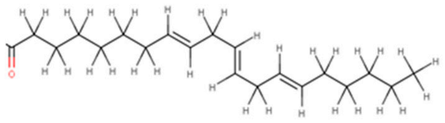

CID-5282826

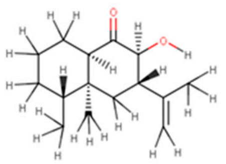

CID-75953512

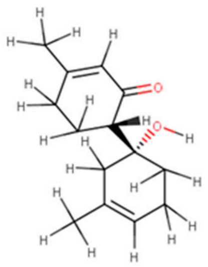

CID-557446

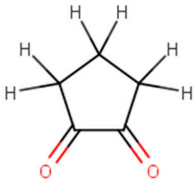

CID-566657

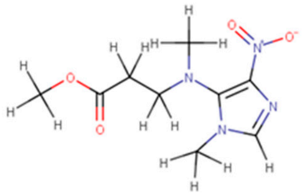

CID-588198

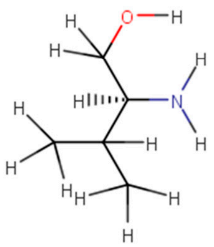

CID-640993

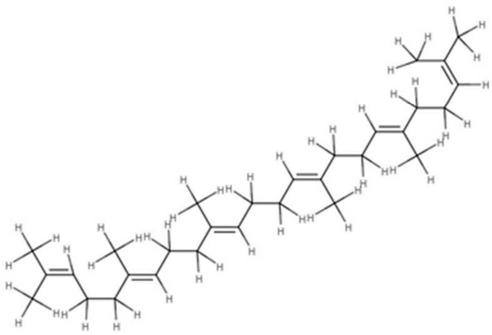

CID-638072

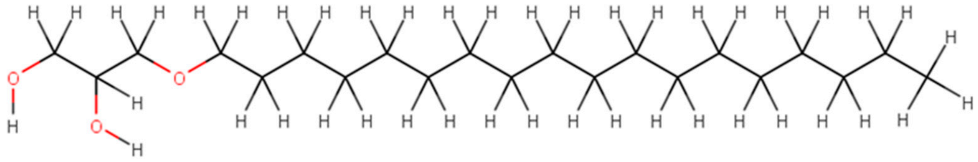

CID-3681

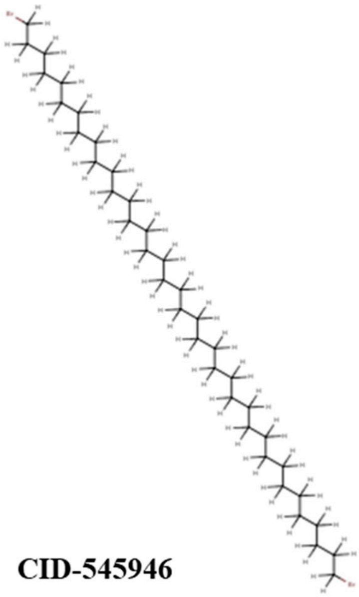

CID-545946

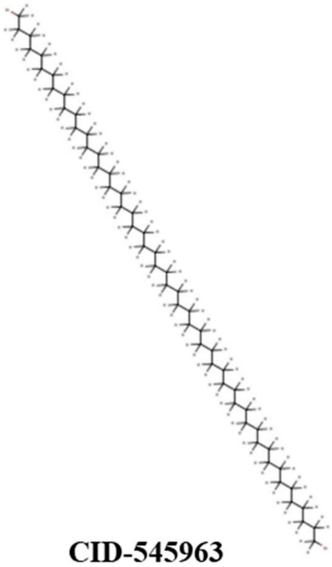

CID-545963

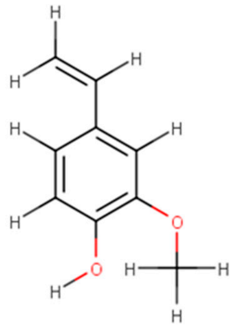

CID-332

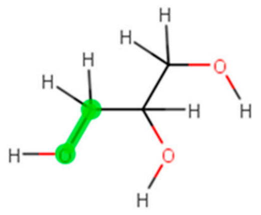

CID-753

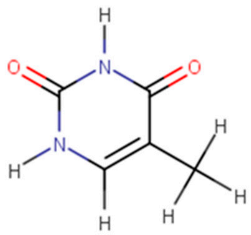

CID-1135

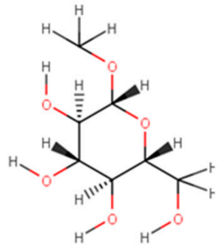

CID-2108

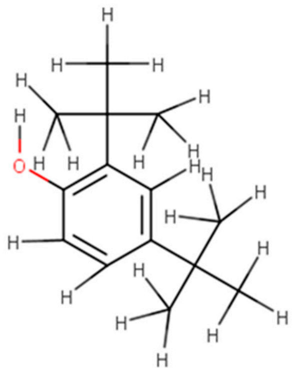

CID-7311

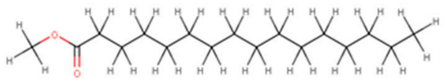

CID-8181

Figure S1: Two dimensional (SD)-structure of *Avicennia officinalis* plant leaf compounds.
